# The Role of Cognitive Behavioral Therapy in the Management of Psychosis

**DOI:** 10.7759/cureus.28884

**Published:** 2022-09-07

**Authors:** Chukwudi Agbor, Garima Kaur, Fareena M Soomro, Victor C Eche, Alexsandra Urhi, Oghenetega E Ayisire, Akinkunmi Kilanko, Funmilola Babalola, Chioma Eze-Njoku, Ngozi J Adaralegbe, Bialo Aladum, Oluwabukola Oyeleye-Adegbite, Gibson O Anugwom

**Affiliations:** 1 Psychiatry, Tees, Esk and Wear Valleys NHS Trust, Scarborough, GBR; 2 Psychiatry, All Indian Institute of Medical Sciences Rishikesh, Rishikesh, IND; 3 Psychiatry and Behavioral Sciences, Isra University, Sindh, PAK; 4 Psychiatry, University of Port Harcourt, Port Harcourt, NGA; 5 Department of Mental Health, Federal Medical Center, Asaba, NGA; 6 Psychiatry, University of South Wales, Pontypridd, GBR; 7 Psychiatry and Behavioral Sciences, La Sierra University, Riverside, USA; 8 Epidemiology and Public Health, Texas Department of State Health Services, San Antonio, USA; 9 Pediatrics, Atrium Health Navicient the Medical Center, Macon, USA; 10 Allied Health Sciences, University of Connecticut, Waterbury, USA; 11 Psychiatry, Ascension Borgess Hospital, Kalamazoo, USA; 12 Department of Public Health, Texas A&M University, College Station, USA; 13 Menninger Department of Psychiatry and Behavioral Sciences, Baylor College of Medicine, Houston, USA

**Keywords:** hospitalisation, cbt for psychosis, management, psychosis, cognitive behavioural therapy

## Abstract

Cognitive behavioral therapy for psychosis (CBTp) as a modality of treatment is gaining attention. A number of authors have reported their experiences, including challenges, in administering CBTp for psychotic patients. With CBTp still evolving a lot more research is ongoing to fine-tune its benefits while mitigating the limitations to its use. The objectives of this review are to determine the role of CBTp in the overall improvement of a patient's quality of life, ascertain the number of hospitalizations with acute symptoms after the start of CBTp; and address the common drawbacks to CBTp in the management of psychosis. It was found that cognitive behavioral therapy (CBT) use can prevent the first episode of psychosis in ultra-high risk (UHR) and is effective in improving depression, self-esteem, and psychological well-being. Its use was associated with positive changes in thinking and mood, and sleep quality leading to improved everyday life. Patients who underwent CBT had fewer hospitalizations with a higher number of voluntary hospitalizations as compared to patients with usual care, who underwent a higher number of involuntary hospitalizations. Drawbacks included cost-ineffectiveness and resource limitation.

## Introduction and background

Psychotic disorders, with a global incidence of 26.6 per 100,000 person-years [1], remain an important healthcare challenge. The attendant morbidity and mortality associated with psychotic disorders make prompt and effective treatment very vital. For decades, pharmacotherapy had been the treatment modality; however other measures either as alternate or adjuvant continue to be evaluated.

Cognitive Behavioral Therapy for Psychosis (CBTp) was initially developed as an individual treatment but was later found to be efficacious in group settings, aimed to reduce the distress associated with the symptoms of psychosis and improve functioning [2,3]. Again, evidence shows CBTp as being effective in preventing transition to full psychosis when applied to those at risk [4]. This study will review the literature on this topic to determine the role of CBTp in the overall improvement of a patient's quality of life and to address the common drawbacks of CBTp in the management of psychosis.

## Review

Methodology

This systematic review was conducted using the preferred reporting items for systematic reviews and meta-analyses (PRISMA). We searched two major databases; EMBASE and PubMed (MEDLINE) using specified search terms. Search terms on the MEDLINE database used are cognitive behavioral therapy or CBT and psychosis. These terms were combined using the Boolean operators (and, or). The search terms used for EMBASE, include (cognitive behavioral therapy/exp or cognitive behavioral therapy or cbt) and (management/exp or management) and of and (psychosis/exp or psychosis). We searched for articles written from January 2012 to April 2022.

Study selection

Studies were selected according to the following criteria:

Inclusion Criteria

Studies that examined and reported the role of Cognitive Behavioural Therapy (CBT) in the overall improvement of the patient's quality of life, studies that xrayed the number of hospitalization with acute symptoms after the start of CBT, and studies that assessed the common drawbacks to CBT in the management of Psychosis, original articles, articles published in January 2012 to April 2022 and articles written in English.

Exclusion Criteria

Articles not written in English, Literature, editorials, and commentaries articles irrelevant to the role of CBT in the management of psychosis. Article before January 2012.

Data collection and study assessment

The authors independently reviewed the abstracts of all the articles identified. Articles adopted were based on the inclusion criteria. The adopted papers were then screened, and a spreadsheet was then created to include all the proposed articles to be used for this research work. All authors were involved in the final selection process.

Data synthesis

**Figure 1:**
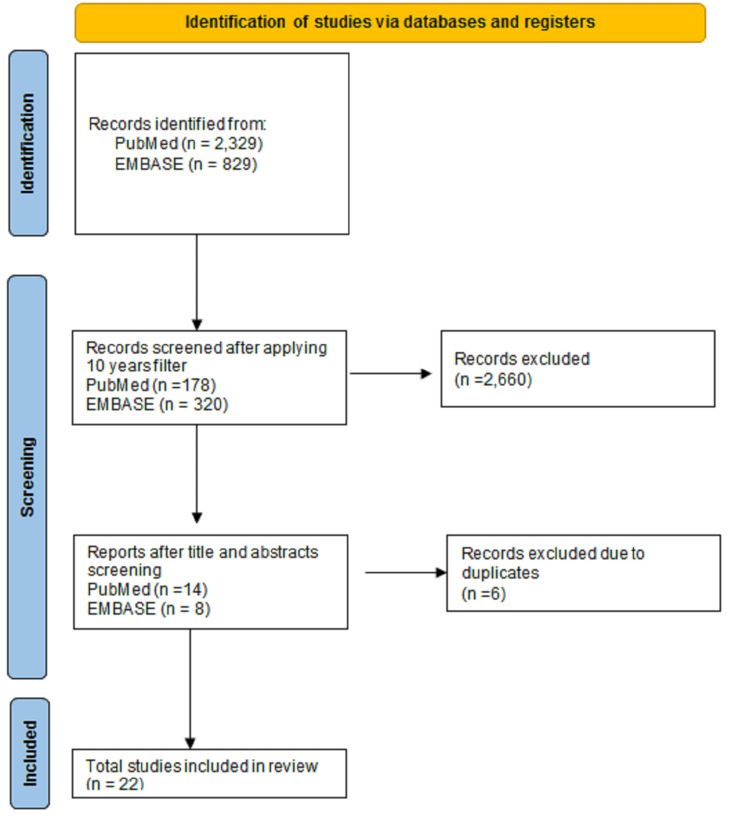
A PRISMA flow diagram showing the Identification and selection of studies used in this review.

The data synthesis was done in a clear and detailed descriptive summary of the included studies via tabulating. The quantitative data were extracted using Microsoft Word. All the identified concepts and themes were arranged and grouped to synthesize significant themes. All authors were responsible for reviewing and discussing major identified themes in the study. Figure 1 demonstrates the literature search process and Table 1 shows the summary of the articles used in this study.

**Table 1:** Summary of articles used in this review.

Author/Year/Title	Study Design	Study Population and characteristics	Sample Size	Summary (Based on the 3 Objectives)
1. Jongsma HE et al., 2017 [1]	Systematic review and meta-analysis.	Individuals with non-organic adult onset psychosis.	-177 .	The global incidence of psychotic disorders is 26.6 per 100000 person-years.
2. Burns AMN et al., 2014 [2]	Meta-analysis	Individuals exhibiting symptoms of psychosis despite adequate trials of medication.	639	CBT is beneficial in the management of medication-resistant psychosis.
3. Wykes T et al., 2022 [3]	Meta-analysis	Individuals suffering from schizophrenia with symptoms.	34 CBTp trials.	CBT can be offered as a group and It is effective in improving symptoms of schizophrenia.
4. Stafford MR et al., 2022 [4]	Systematic review and meta-analysis.	Individuals at risk without a formal diagnosis of bipolar disorder or schizophrenia	11 trials including 1246 participants.	It may be possible to delay or prevent transition to psychosis with CBT in individuals at risk.
5. Van der Gaag M et al., 2019 [5]	Systematic review	Patients with ‘at-risk mental state’ (ARMS)		CBTp can be used in at-risk mental state patients to prevent transition to first episode psychosis. More effective in this group because they still have ‘insight’ as compared to frank psychosis
6. Ising HK et al., 2017 [6]	Randomised control trial	-14-35 years - Family history of psychosis or Comprehensive Assessment of At-Risk Mental States (CAARMS).	196	CBTuhr had an 83% likelihood of resulting in a reduction of the transition to psychosis at a lower cost.
7. Morrison AP et al., 2019 [7]		People with Clozapine Resistant Schizophrenia aged ≥ 16 years, with an ICD-10 schizophrenia spectrum diagnoses and who are experiencing psychotic symptoms.	487 [242 were allocated to CBT + TAU and 245 to TAU(Treatment as Usual)]	-Cognitive behavioural therapy for CRS was not found superior to TAU at 21 months, but was superior at 9 months (end of treatment). -CBT resulted in improved quality of life with a net QALY gain of 0.052 compared with the usual treatment -Study reported that there was no suggestion that the addition of CBT to TAU caused adverse effects. CGI group reported 33 voluntary and 10 involuntary hospitalizations while TAU reported 24 and 14 respectively. -CBT was not found to be cost-effective in comparison with TAU.
8. Wijnen BFM et al., 2018 [8]	Randomised control trial	Single-blinded randomized control trial between treatment-as-usual group versus treatment-as-usual augmented with adjunct CBTsa	50 participants in treatment-as-usual group and 49 participants in treatment -as-usual with adjunctive CBTsa	Found higher costs as compared to treatment-as-usual, as much as 20000-80000euros to achieve 1 QALY
9. Sheaves B et al., 2019 [9]	Randomized control trial	NA	-feasibility outcomes assessed therapy uptake, techniques used, satisfaction, and attrition. -The primary efficacy outcome assessed nightmare severity at week	-CBT for nightmares is feasible and may be efficacious for treating nightmares and comorbid insomnia for patients with persecutory delusions. -It shows promise on paranoia but potentially not on suicidal ideation.
10. Liu Y et al., 2019 [10]	Randomized control trial	-Participants recruited from Beijing Anding hospital affiliate between June 2012 and march 2014. -Participants randomized to a CBTp plus treatment-as-usual group or just a treatment-as-usual group	80	Brief CBTp targeting positive symptoms was effective in first-episode schizophrenia
11. Waller H et al., 2015 [11]	Randomized controlled trial	Participants with persistent, stable persecutory delusions were recruited from adult community mental health teams		-In CBT arm the participants underwent the Thinking Well intervention in which they first completed the Maudsley Review Training Programme (MRTP) followed by four individualised CBT sessions. -Clinically beneficial effects were seen for belief flexibility, state paranoia and on distress and conviction.
12. Muller H et al., 2020 [12]	Randomized controlled trial	Adolescents with early onset psychosis	25 at the start (CBT +TAU =13, TAU=12) Lost to follow up=9	-There were no statistically significant differences between the two treatment arms post-treatment on any of the psychopathology, functional, remission measures but , CBT+TAU showed a significantly higher score on the “material quality of life” subscale -There were seven hospital admissions during the time of intervention: only two of them belonged to the CBT+TAU group, whereas five participants of TAU were hospitalized.
13. Freeman D et al., 2014 [13]	Randomized controlled trial	Patients with current persecutory delusions	30 (15 were allocated to CBT + standard care and 15 to standard care)	-The study reported improvement in psychological well-being, positive beliefs about the self, negative social comparison, self-esteem, and depression. -Post treatment there was a small reduction in negative self-beliefs and a moderate reduction in paranoia but these were not statistically significant.
14. Sönmez N et al., 2019 [14]	Randomized control trial	Early psychosis patients were included and randomly assigned to receive either CBT (maximum 26 sessions) or TAU for a period of up to six months.	63	They did not find CBT to be more effective than TAU in reducing depressive symptoms or increasing self-esteem in patients with early psychosis. However, CBT seems to improve negative symptoms and functioning.
15. Granholm E et al., 2014 [15]	Randomized control trial	-Participants were recruited through flyers and brochures posted and handed out by a study recruiter at a variety of community	149 .	The results suggest CBSST is an effective treatment to improve functioning and experiential negative symptoms in consumers with schizophrenia, and both CBSST and supportive group therapy actively focused on setting and achieving functioning goals can improve social competence and reduce positive symptoms.
16. Fitriani N et al., 2021 [16]	Quasi-experimental without control group. 4 sessions of CBT and 5 sessions of social skill training , using patients’ schizophrenia workbooks and diaries	74 patients with schizophrenia.	30 patients were sampled using consecutive sampling technique	Combination of CBT and SST has positive changes in the sign and symptoms of risk for violent behaviour patients with schizophrenia, and these changes impact the ability of patients to control their symptoms
17. Brabban A et al., 2022 [17]	Randomized control trial	Patients aged 18-65 years who had a formal diagnosis of Schizophrenia and who were receiving care from secondary mental health services.	354 .	-CBT improves psychosis in Schizophrenia. -Being female led to a 25% increase in Insight and reduction in overall symptoms following CBT
18. Xanidis N et al., 2020 [18]	Qualitative study with focus groups and individual interviews	Members of staff working in the community and crisis mental health teams. Mental health nurses (n = 5), consultant psychiatrists (n = 2), clinical/counselling psychologists (n = 2), CBT therapists (n = 2), an occupational therapist (n = 1), a team leader (n = 1), and a senior adult mental health manager (n = 1).	The results of this study suggested a mixture of barriers and facilitators to CBTp implementation.	-Challenges that physicians face while applying CBTp are the intensity of symptoms and reported lack of insight of patients with psychosis. Furthermore, difficulties with consistent attendance enhanced experts' pessimism about the feasibility of recovery and reinforced the lack of implementation. -In addition, Professionals are unable to apply CBTp due to a high caseload, a lack of protected time, and supervision.
19. Kopelovich SL et al., 2019 [19]	Prospective study with administration of a learning collaborative (LC) model and a biweekly phone-based follow-up over a six- month period. Additional six month follow-up for 21 of the persons after phase 1	psychotic volunteers from 12 different agencies across Florida state	56	-Low implementation of CBTp due to resource limitation. Recommended brief CBTp targeting specific symptoms as a measure to address this challenge. Also noted reduction in relapse with brief CBTp
20. Grossi LM et al., 2021 [20]	Systematic review	NA	NA	-CBTp can be used to restore and ensure durability of competency in psychotic suspects. -It can be used in traditional treatment-refractory cases in the face of compliance with medications.
21. Dunn G et al., 2012 [21]	Randomized control trial	NA	133	-Consistent delivery of full therapy, including specific cognitive and behavioural techniques, was associated with clinically and statistically significant increases in months in remission, and decreases in psychotic and affective symptoms. -CBT-P is of significant benefit on multiple outcomes to patients able to engage in the full range of therapy procedures. The novel statistical methods illustrated in this report have general application to the evaluation of heterogeneity in the effects of treatment.

CBT and prevention of first episode psychosis

One of the striking uses of CBT is its use in preventing first-episode psychosis in individuals with ultra-high risk (UHR). A study done by Van der Gaag et al. [5] observed similar results and attributed the success of CBT in this group to the presence of insight as opposed to patients with frank psychoses. In fact, Helga et al. reported an 83% likelihood of a reduction in the transition to first episode psychosis and a 75% likelihood of more quality-adjusted life year (QALY), equivalent to one year of life in perfect health, gains at lower costs, and these effects were sustained for four years [6]. The role of CBT in UHR individuals was further illustrated by the finding of a higher remission rate in UHR symptomatology following CBT.

Overview of CBT and psychosis

A protocol was written by French and Morrison from the Early Detection and Intervention Evaluation (EDIE) trial: a randomized control trial with 60 patients [7]. The CBT intervention is based on a formulation-driven cognitive model that prioritizes a collaboratively agreed problem list. It is a problem-oriented, time-limited, and educational treatment, using collaborative empiricism with guided discovery, behavioral experiments, and homework tasks. The model draws on strategies for change, including normalization, generating, and evaluating alternative beliefs, safety behaviors, meta cognitions, core beliefs, social isolation, and relapse prevention. The strategies used were selected in accordance with the formulation and key problems identified on the participant's problem list [7]. Post-treatment, eight of the nine assessment variables showed signs of improvement in treatment, ranging from small to large effects. Five out of nine post-treatment tests reached statistical significance, with the treatment group benefiting in each case [7]. For the primary measures, there was a small effect size reduction in negative beliefs about the self and a moderate effect size reduction in paranoia, neither of which was statistically significant. For the statistically significant secondary outcomes, there were notable improvements in psychological well-being, positive beliefs about the self, self-esteem, social comparison, and depression. Anxiety levels were slightly lower in the control group post-treatment. No benefits of treatment were maintained at the 12-week assessment [7].

The role of CBT in schizophrenia

CBT has been shown to affect both positive and negative symptoms of schizophrenia, some areas of cognition, lowering negative symptoms, and improvement in attention and memory [5,8], and were mainly seen in those with better attendance. It is safe to assume then that long-term CBT has better effects than short-term. The role of CBT in suicidal ideation was also explored by the Sheaves et al., study which suggested that it has some promising effects on paranoia [9]. Yan Liu et al. reported that in patients with first-episode schizophrenia, brief CBTp targeting positive symptoms was shown to lead to improvement in these symptoms [10]. 

CBT and quality of life

In a randomized control trial done by Waller et al. [5] in which participants underwent: Thinking well intervention [A Maudsley Review Training Programme (MRTP) followed by four individualized CBT sessions] beneficial effects were noted for belief flexibility, state paranoia, distress, and conviction. Participants also reported that the intervention led to positive changes in thinking and mood, resulting in improved everyday life. However, these results were not fully sustained at the six weeks follow-up [11]. In another study, significant improvement was reported in “material quality of life” in patients undergoing CBT compared to treatment per usual [12]. There was an improvement in sleep following a targeted CBT called CBT for insomnia in patients with persistent delusions and hallucinations [11]. Sheaves et al. also noted similar findings in their study on insomnia and sleep in a similar population [9]. Hence, improving their quality of life.

CBT and delusions

In individuals with persecutory delusions, CBT has been demonstrated to enhance self-esteem and psychological well-being, and improve depression. It is suggested that it could lead to a decrease in persecutory delusions [13]. While CBT seems to improve negative symptoms and functioning, Sonmez et al. did not find it more effective than treatment as usual in reducing depressive symptoms or increasing self-esteem in patients with early psychosis [14]. This was supported by another study done a year later, where they noted similar observations [14]. Morrison et al. discovered that CBT in addition to usual therapy was superior in terms of improvement in positive and negative symptoms in patients with clozapine-resistant schizophrenia at the conclusion of treatment, with a net QALY gain of 0.052 years [7]. The effect, however, did not remain at the one-year follow-up, although the improvement in emotional distress caused by delusions was effectively sustained [7].

Combined effect of cognitive behavioral therapy and cognitive behavioral social skills training

Social skills deficit can affect several aspects of health and social interaction. Our review found that CBT can be combined with social skills training, a combination called cognitive behavioral social skills training (CBSST), in the management of Schizophrenia. Eric Granholm et al. noted that CBSST led to a reduction in negative and positive symptoms while increasing competence and social functioning in individuals who suffer from schizophrenia [15]. Nurlaila Fitriani et al., further, discovered that CBSST resulted in favorable changes in the signs and symptoms of patients with schizophrenia who are at risk for violent behavior [16], these changes enhanced patients' understanding of their symptoms and improved their ability to control their behavior [16]. However, the lack of a control group to compare the effectiveness of the intervention was a weakness of their study and justifies additional research in this aspect [16].

CBT and hospitalizations

We further explored the effect of CBT on hospitalizations. Müller et al. found that patients in the CBT plus treatment as the usual group had fewer hospitalizations than those in the usual care group [12]. Another study indicated that patients who received CBT had a higher number of voluntary hospitalizations, but those who received standard care had a larger number of involuntary hospitalizations [12]. This raises the question of whether CBT actually improves cognition in these patients who are thus amenable to voluntary hospitalizations.

Factors influencing outcomes of CBT

Sex, level of delusion convictions, and a greater number of hospitalizations seem to be a mitigating factor with regards to the effect of CBT. Brabban et al. discovered that being female and having a minimal level of conviction in delusions were associated with better outcomes [17].

Challenges in CBT delivery

Having reviewed the positive impact of CBT in patients with psychosis we further explored the challenges that physicians and patients face in the implementation of CBT. Xanidis et al. reported severe intensity of symptoms and lack of insight in patients with psychosis are important limitations faced by physicians [18]. Furthermore, due to a large caseload, a lack of protected time, and supervision, professionals are unable to use CBTp. Difficulties with consistent attendance were noted to increase expert's pessimism regarding the feasibility of recovery which reinforced the lack of implementation of CBT. Peer and family support were identified as a perceived barrier to the engagement of patients with CBT. The findings within a Normalisation process Theory framework noted the necessity of strong clinical leadership in addressing issues related to its implementation [18]. This would involve training the current workforce, and ensuring protected time defined in job roles for delivery, in addition to increasing other professionals’ awareness of the nature and purpose of CBTp.

In a prospective study, Kopelovich et al. identified resource limitation as one of the reasons for the low implementation of CBTp [19]. To address this difficulty, brief CBTp focusing on specific symptoms was recommended [19]. Brief CBTp was also found to reduce relapse [20]. A key drawback in CBTp implementation is its low likelihood to be cost-effective. In a study done in the Netherlands, Wijnen et al. discovered that patients who received CBT plus usual care had greater expenses in services, ranging from 20000-80000 euros (21000-85000 dollars) per QALY gained, as compared to patients who received usual care alone [8].

Throughout our review, we found out that consistent delivery of CBT is key in achieving and maintaining desired results. For example, Dunn G et al., observed that consistent administration of CBT was associated with clinically and statistically significant increases in months in remission and decreases in psychotic and affective symptoms [21]. However, there are several challenges to the continuous administration of CBTp. Firstly, CBTp is complex and its effective administration depends on the interaction between therapist and patient, hence, factors such as readiness and willingness of the patient, nature of symptoms, and awareness level of distress on the part of the patient can influence the overall result. Additionally, the ability, training, supervision, adherence, and competence of the trainer are also key factors in achieving desired results.

## Conclusions

The findings in this systematic review suggest that CBT in addition to standard care in the management of psychotic symptoms leads to improved quality of life. CBT leads to a decrease in psychotic and affective symptoms and improves functioning. It was found that CBT is particularly effective in preventing first-episode psychosis in high-risk individuals and in improving depression, self-esteem, and psychological well-being in individuals with persecutory delusions. It has also been found to be effective in Clozapine resistant schizophrenia. Patients who received CBT had fewer hospitalizations than those who received usual care. Consistent delivery of CBT is found to be the key to achieving and maintaining desired results. There are several challenges to the continuous administration of CBT for psychosis including resource limitation, Lack of insight, high costs, and a lack of protected time for its administration.
